# REMOTE FAMILY CAREGIVERS: INVISIBLE YET INDISPENSABLE DURING HOSPITALIZATION OF OLDER ADULTS

**DOI:** 10.1093/geroni/igad104.3571

**Published:** 2023-12-21

**Authors:** Orly Tonkikh, Yuuko Johnson, Heather Young, Elena O Siegel

**Affiliations:** University of California Davis, Sacramento, California, United States; University of California Davis, Sacramento, California, United States; University of California Davis, Sacramento, California, United States; University of California Davis, Sacramento, California, United States

## Abstract

Limited research has addressed the interplay between the roles of nursing staff and families of older adults in acute care settings, including those families involved in-person and those who are unable to be physically present (remote). This descriptive qualitative study explores nurses’ and family members’ perspectives on the roles and engaging family members or friends during an acute hospitalization in medical-surgical units. Semi-structured interviews were conducted via Zoom or phone with a convenience sample of 19 nurses and families between February and August 2023. Thematic analysis inductively captured key patterns in data. Nurses proactively engaged family members who were present in the hospital, while they engaged with remote family members in a more limited way, mostly during discharge planning. Nurses describe making decisions about including families based on their presence (e.g., education about medications, encouraging to engage the patient in the recommended care, ask to support with ADLs). In contrast, families describe providing support regardless of physical presence, including socio-emotional support, help with medical/nursing tasks, encouragement to recover, mediation of information provided by staff, learning to perform medical-nursing tasks and communication and interpretation of the information with other family members. The findings reveal a gap in nurses and family’s perspectives about the feasibility and contribution of remote family involvement during an acute hospitalization. This provides direction for further exploration of acute care structures and nursing care processes needed to support the preferred mode for family involvement.

